# Prolyl 4-hydroxylase subunit beta (P4HB) could serve as a prognostic and radiosensitivity biomarker for prostate cancer patients

**DOI:** 10.1186/s40001-023-01215-2

**Published:** 2023-07-22

**Authors:** Dechao Feng, Li Li, Dengxiong Li, Ruicheng Wu, Weizhen Zhu, Jie Wang, Luxia Ye, Ping Han

**Affiliations:** 1 Department of Urology, Institute of Urology, West China Hospital, Sichuan University, Guoxue Xiang #37, Chengdu, 610041 Sichuan People’s Republic of China; 2 Department of Public Research Platform, Taizhou Hospital of Zhejiang Province Affiliated to Wenzhou Medical University, Linhai, China

**Keywords:** Prolyl 4-hydroxylase subunit beta, Prostate cancer, Radiotherapy resistance, Cell proliferation, Ferroptosis

## Abstract

**Background:**

Prolyl 4-hydroxylase subunit beta (P4HB) has been reported as a suppressor in ferroptosis. However, no known empirical research has focused on exploring relationships between P4HB and prostate cancer (PCa). In this research, we initially examine the function of P4HB in PCa by thorough analysis of numerous databases and proliferation experiment.

**Methods:**

We analyzed the correlations of P4HB expression with prognosis, clinical features, mutation genes, tumor heterogeneity, stemness, tumor immune microenvironment and PCa cells using multiple databases and in vitro experiment with R 3.6.3 software and its suitable packages.

**Results:**

P4HB was significantly upregulated in tumor tissues compared to normal tissues and was closely related to biochemical recurrence-free survival. In terms of clinical correlations, we found that higher P4HB expression was significantly related to older age, higher Gleason score, advanced T stage and residual tumor**.** Surprisingly, P4HB had highly diagnostic accuracy of radiotherapy resistance (AUC 0.938). TGF beta signaling pathway and dorso ventral axis formation were upregulated in the group of low-expression P4HB. For tumor stemness, P4HB expression was positively related to EREG.EXPss and RNAss, but was negatively associated with ENHss and DNAss with statistical significance. For tumor heterogeneity, P4HB expression was positively related to MATH, but was negatively associated with tumor ploidy and microsatellite instability. For the overall assessment of TME, we observed that P4HB expression was negatively associated with all parameters, including B cells, CD4+ T cells, CD8+ T cells, neutrophils, macrophages, dendritic cells, stromal score, immune score and ESTIMATE score. Spearman analysis showed that P4HB expression was negatively related to TIDE score with statistical significance. In vitro experiment, RT-qPCR and western blot showed that three siRNAs of P4HB were effective on the knockdown of P4HB expression. Furthermore, we observed that the downregulation of P4HB had significant influence on the cell proliferation of six PCa cell lines, including LNCap, C4-2, C4-2B, PC3, DU145 and 22RV1 cells.

**Conclusions:**

In this study, we found that P4HB might serve as a prognostic biomarker and predict radiotherapy resistance for PCa patients. Downregulation of P4HB expression could inhibit the cell proliferation of PCa cells.

## Introduction

In 2022, new cancer cases are projected to total 4,820,000 and 2,370,000 in China and the USA, respectively [1]. Prostate cancer (PCa) serves as the third and sixth most prevailing malignancy among newly diagnosed cases in China and the USA, respectively [1–3]. The incidence, mortality, and disability-adjusted life years for PCa were also greater in areas and nations with higher sociodemographic indices [4]. Inflammation and age are risk factors of many diseases, such as macular degeneration [5, 6], cardiovascular diseases [7, 8], periodontitis [9], neurological disorders [10], and human cancer [11–15]. Globally, aging is posing a severe threat to human health [16]. The vast majority of PCa cases occurred in elderly people and it is anticipated that this trend will worsen as the world’s population ages [4, 17–24]. There is growing recognition that genetic variability in PCa encompasses many tumor forms with unique biologic characteristics and clinical behaviors [25]. The primary methods for treating patients with localized PCa include radical prostatectomy or radiation, while biochemical recurrence (BCR) is unavoidable for patients after radical prostatectomy (27–53%) or radiotherapy (10–70%), respectively [26–28]. Eight years is the typical period from BCR to metastasis, while 5 years is the median time from metastatic to death [19, 27, 28]. In addition, treatment resistance is fast developing more deadly and malignant neuroendocrine PCa, and the prognosis for such patients is quite dismal with a survival time of less than 1 year [29]. By integrating genetic and clinical data, the advancement of sequencing technology, particularly the completion of the Cancer Genome Atlas (TCGA), offers a feasible and effective method to screen such patients.

The role of ferroptosis has received increased attention across a number of diseases, since it was proposed by Prof. Brent R.Stockwell et al. in 2012 [30]. It is a novel of nonapoptotic cell death and is controlled by iron pool, lipid metabolism and antioxidant metabolism [31–34]. In 2020, Dr. Nan Zhou and Jinku Bao proclaimed an excellent FerrDb data set, which took 784 ferroptosis studies from the PubMed database and extracted ferroptosis regulators and markers and relevant diseases [35]. Using the TCGA and FerrDb databases [35], prolyl 4-hydroxylase subunit beta (P4HB) and prostaglandin–endoperoxide synthase 2 (PTGS2) were detected to be potential biomarker for PCa patients from the perspective of ferroptosis, where research to date has not yet determined the impact of P4HB on PCa patients. Herein, through a thorough review of numerous databases and an in vitro experiment, we primarily investigated the function of P4HB in PCa.

## Methods

### Bioinformatic analysis

We obtained the PCa data of TCGA database and gene expression omnibus (GEO) data sets (GSE46602 [36], GSE32571 [37] and GSE62872 [38]) from our previous study [19]. From the FerrDb database, 474 genes relevant to human ferroptosis were taken [35]. llogFCl 0.5 and p.adj 0.05 were used to determine the differentially expressed genes (DEGs) between 498 tumour and 52 normal samples in the TCCG database. The BCR-free survival P value was constrained to less than 0.05. By combining DEGs, prognosis-related genes, and ferroptosis-related genes, P4HB and PTGS2 were discovered. GEO data sets [36–38] were utilized to confirm the differential expression of P4HB and PTGS2. We selected P4HB for further research due to its less information in PCa. The pan-cancer distinct expression of P4HB between cancer and normal samples was evaluated through the TIMER database [39]. In addition, we again confirmed P4HB expression between samples of cancer and normal using UALCAN [40] and GEPIA [41]. We then conducted an analysis of the clinical P4HB levels and created a nomogram. Using GSE53902 [42], We evaluated P4HB's diagnostic effectiveness with regard to radiation resistance. Mutation data of PCa patients were downloaded from GDC (https://portal.gdc.cancer.gov/) and were visualized by MuTect2 software and R package “maftools (version 2.2.10)” [43]. Using the median value of P4HB expression, we divided the PCa patients into groups based on the expression levels. Analysis was done on the variations about gene mutation frequency between these two groups. We used the human protein atlas (HPA) to examine P4HB's potential intracellular localization [44, 45]. We also assessed the predicted functional partners of P4HB using multiple databases, including HPA, GeneMANIA [46] and STRING [47].

In terms of the functional analysis, We used the R program "gene set variation analysis (GSVA)" [48] and “c2.cp.v7.4.symbols.gmt” subset from the molecular signature database [49] to determine the enrichment scores of each sample's relevant molecular processes and pathways. The set contained between 5 and 5000 genes. The "wilcox.test" tool was then applied to compare each pathway between P4HB expression levels that were high and low. The fold change was 1.5, and statistical significance was defined as p. adj. 0.01 and false discovery rate 0.01. Using the Spearman analysis, the overall tumor immune microenvironment (TME) and immune cells were evaluated by ESTIMATE and TIMER algorithms [50–52]. The TISIDB database examined the interactions of P4HB with tumor-infiltrating cells, immunoinhibitors, and immunostimulators [53]. Poor effectiveness of immune checkpoint blockade (ICB) is associated with high tumor immune dysfunction and exclusion (TIDE) score. We used the TIDE algorithm [54] to estimate the TIDE score and assess the relationship of P4HB with TIDE score with Spearman analysis.

The correlation between P4HB and tumor heterogeneity and stemness related indexes was analyzed and the specific methods could be seen from the previous studies [55–62]. GSCALite was used to examine the relationship between P4HB and medication sensitivity in pan cancer [63], which integrated the data of the cancer therapeutics response portal and genomics of drug sensitivity in cancer.

### In vitro* experiment*

**Fig. 1:**
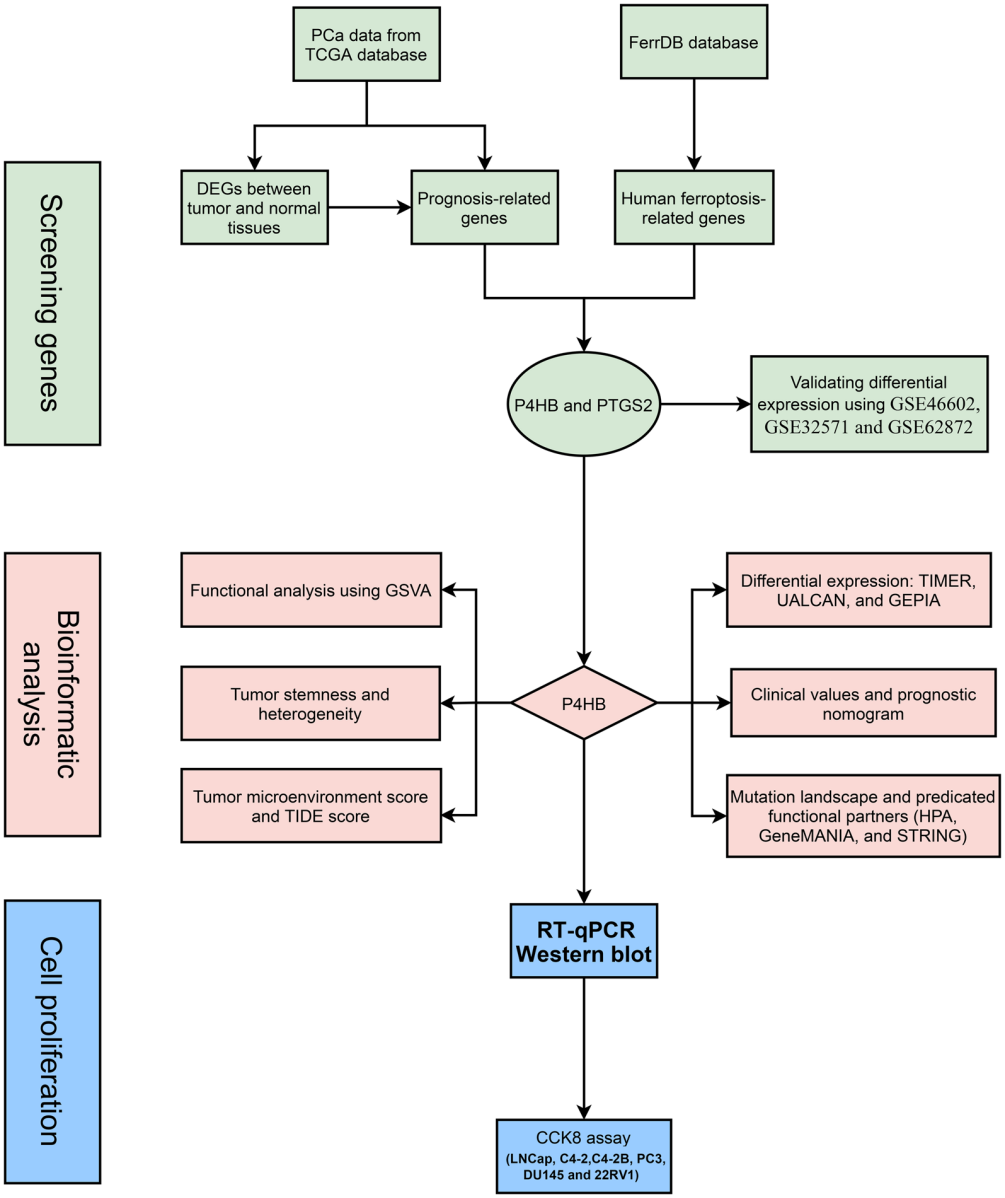
Flowchart of this study. *Pca* prostate cancer, *DEGs* differentially expressed genes, *GSVA* gene set variation analysis, *TIDE* tumor immune dysfunction and exclusion, *RT-qPCR* real-time quantitative polymerase chain reaction, *TCGA* the cancer genome atlas

The acquisition of PCa cell lines and their culture were described from our previous study [64]. The culture condition of 22RV1 cell is similar to common PCa cells, such as DU145 cells. In addition, RT-qPCR methods was also described in the previous study [64]. siRNA was obtained by HIPPOBIO (www.hippobiotec.com). P4HB si-1sense: 5′-UGCUGUUCUUGCCCAAGAGUGdTdT-3′; P4HB si-1 antisense: 5′-CACUCUUGGGCAAGAACAGCAdTdT-3′. P4HB si-2 sense: 5′-AGGUGAAAUCAAGACUCACAUdTdT-3′; P4HB si-2 antisense: 5′-AUGUGAGUCUUGAUUUCACCUdTdT-3′; P4HB si-3 sense: 5′-GUGUGGUCACUGCAAACAGUUdTdT-3′; P4HB si-3 antisense: 5′-AACUGUUUGCAGUGACCACACdTdT-3′. An internal control was implemented using glyceraldehyde-3-phosphate dehydrogenase (GAPDH). GAPDH: 5′-CTGGGCTACACTGAGCACC-3′ (forward) and 5′-TCC AAGTGGTCGTTGAGGGCAATG-3′ (reverse); P4HB: 5′-GGTGCTGCGGAAAAGCAAC-3′ (forward) and 5′-ACCTGATCTCGGAACCTTCTG-3′ (reverse). The siRNAs of P4HB that were most efficient were found using RT-qPCR. Cells were collected and lysed in RIPA Lysis Buffer containing freshly added PMSF and a protease inhibitor cocktail for the western blot. Using a BSA kit, protein concentration was discovered. A 30 ug protein sample was run via an SDS–PAGE gel. Gel electrophoresis was used to separate the proteins, which were then transferred to a polyvinylidene difluoride membrane. The membranes were blocked for 2 h at room temperature with 5% nonfat dry skim milk before being incubated with matching primary antibodies overnight at 4 ℃. The main antibodies are anti-P4HB (1:1000, ab2792 Abcam, USA) and β-Actin (1:10,000, 81115-1-RR, Proteintech, China). The membranes were detected using horseradish peroxidase-conjugated secondary antibodies (1:3000, SA00001-1, SA00001-2, Proteintech, China) followed by exposure to enhanced chemiluminescence substrate (Millipore, WBKLS0500, USA). Six PCa cell lines were transfected with P4HB siRNAs, and the impact of P4HB on their ability to proliferate was examined using the cell counting kit-8 (CCK8) assay at 24 h, 48 h, and 72 h. The study's flowchart is shown in Fig. 1.

### Statistical analysis

For statistical analysis, R 3.6.3 software and its appropriate packages were utilised. Through the use of log-rank tests and Kaplan–Meier curves, the survival analysis was carried out. Based on the findings of the Cox regression studies, a nomogram was created, and the nomogram model was assessed using the Harrell's concordance index (C-index) and decision curve analysis (DCA) curve. The aforementioned statistical tests are all two-sided. Statistical significance was defined as a *P* value of 0.05. The following symbols were noteworthy: ns, *p* ≥ 0.05; *p < 0.05; **p < 0.01; ***p < 0.001.

## Results

### Bioinformatic analysis

**Fig. 2:**
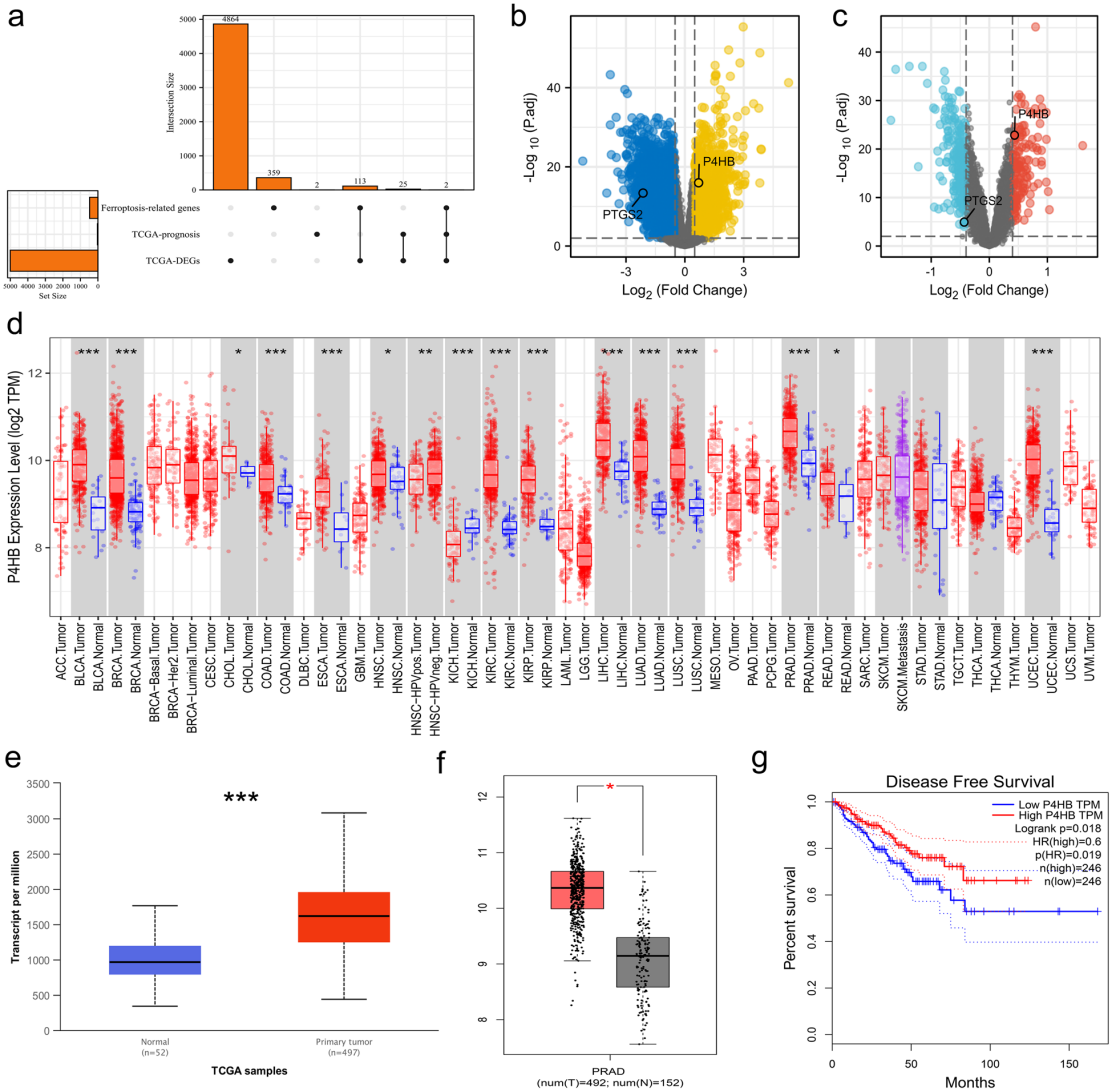
Process of screening P4HB. **a** Upset plot shwnig the intersection of DEGs, prognosis-related genes and ferroptosis-related genes; **b** volcano plot showing the expression of P4HB and PTGS2 between tumor and normal samples in the TCGA database; **c** volcano plot showing the expression of P4HB and PTGS2 between tumor and normal samples in the GEO data sets; **d** bar graph showing the expression of P4HB between tumor and normal samples at pan-cancer level; **e** bar graph showing P4HB expression between tumor and normal samples in the UALCAN database; **f** bar graph showing P4HB expression between tumor and normal samples in the GEPIA database; **g** Kaplan–Meier curve showing the survival differences of high and low P4HB expression using GEPIA database. *DEGs *differentially expressed genes, *TCGA* the cancer genome atlas, *GEO* gene expression omnibus

**Fig. 3:**
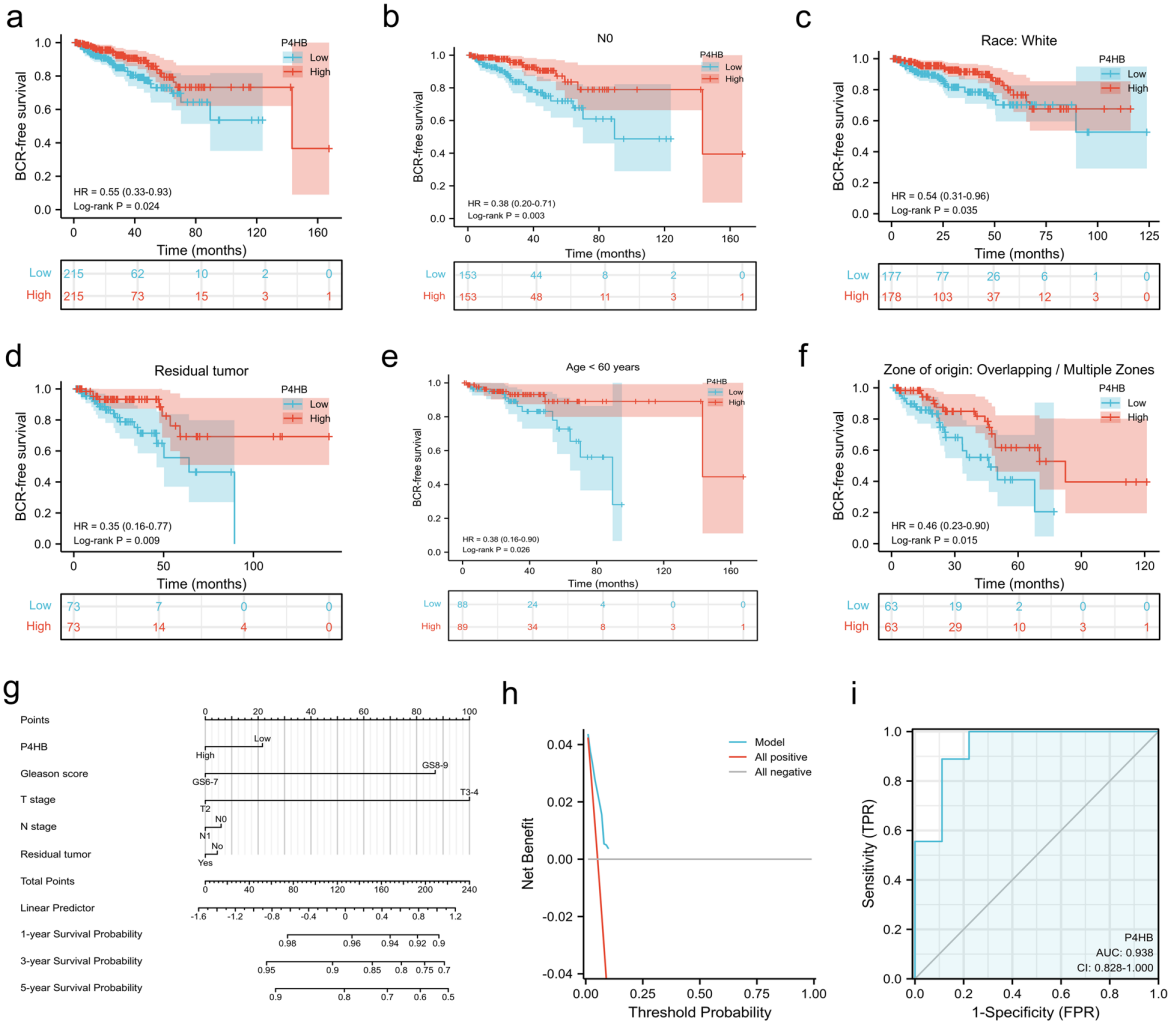
Correlations of P4HB with prognosis and radiotherapy resistance. **a** Kaplan–Meier curve showing the survival differences of high and low P4HB expression in the TCGA database; **b** Kaplan–Meier curve showing the survival differences of high and low P4HB expression in patients with N0 stage; **c** Kaplan–Meier curve showing the survival differences of high and low P4HB expression in White patients; **d** Kaplan–Meier curve showing the survival differences of high and low P4HB expression in patients with residual tumor; **e** Kaplan–Meier curve showing the survival differences of high and low P4HB expression in patients with age < 60 years; **f** Kaplan–Meier curve showing the survival differences of high and low P4HB expression in patients with overlapping or multiple zones; **g** nomogram plot integrating P4HB expression and clinical featurs; **h** decision curve analysis curve; **i** ROC curve showing the diagnostic ability of P4HB in radiotherapy resistance. *TCGA* the cancer genome atlas, *ROC* receiver operating characteristic curve

P4HB and PTGS2 were discovered as a result of the intersection of DEGs, prognosis-related genes, and ferroptosis-related genes (Fig. 2a). P4HB was upregulated in the TCGA database (Fig. 2b) and was validated in the GEO data sets [14–16] (Fig. 2c). With statistical significance, P4HB was differently expressed in a variety of malignancies, including PCa (Fig. 2d). In comparison to normal samples, UALCAN [18] and GEPIA [19] were used to confirm that tumor samples had increased P4HB expression (Fig. 2e, f). In the GEPIA database [19], Based on the median value of P4HB, PCa patients were split into two groups, and those with lower expression of P4HB had significantly shorter disease-free life than those with higher expression of P4HB (Fig. 2g). In addition, we found that P4HB was strongly linked with BCR-free survival in our analysis (Fig. 3a). In the subgroup survival study, higher P4HB expression was associated with a reduced probability of BCR in terms of N0 stage, White population, residual tumor, age < 60, and overlapping or multiple zones than lower P4HB expression (Fig. 3b–f). In addition, we created a nomogram incorporating P4HB and clinical characteristics to forecast the likelihood of BCR for PCa patients (Fig. 3g). DCA curve indicated that this might be acceptable (C-index: 0.718; Fig. 3h). Surprisingly, P4HB demonstrated a high level of diagnostic accuracy about radiation resistance (AUC: 0.938; Fig. 3i). Clinical correlations revealed that older age, a higher Gleason score, an advanced T stage, and residual tumour were all strongly correlated with increased P4HB expression (Table 1).

**Table 1:** Clinical features between high and low expressions of P4HB for prostate cancer patients in the TCGA database

Characteristics	P4HB expression		*P* value
	Low	High	
Sample	215	215	
Age, median (IQR)	60 (55, 65)	62 (57, 67)	0.009
Gleason score, *n* (%)			< 0.001
GS6	24 (5.6%)	15 (3.5%)	
GS7	122 (28.4%)	84 (19.5%)	
GS8	27 (6.3%)	32 (7.4%)	
GS9	42 (9.8%)	84 (19.5%)	
T stage, *n* (%)			0.002
T2	92 (21.7%)	63 (14.9%)	
T3	118 (27.8%)	143 (33.7%)	
T4	1 (0.2%)	7 (1.7%)	
Race, *n* (%)			0.271
Asian	8 (1.9%)	3 (0.7%)	
Black or African American	23 (5.5%)	27 (6.5%)	
White	176 (42.3%)	179 (43%)	
N stage, *n* (%)			0.596
N0	151 (40.3%)	155 (41.3%)	
N1	31 (8.3%)	38 (10.1%)	
Residual tumor, *n* (%)			0.008
No	149 (35.6%)	124 (29.6%)	
Yes	59 (14.1%)	87 (20.8%)	

*IQR* interquartile range

**Fig. 4:**
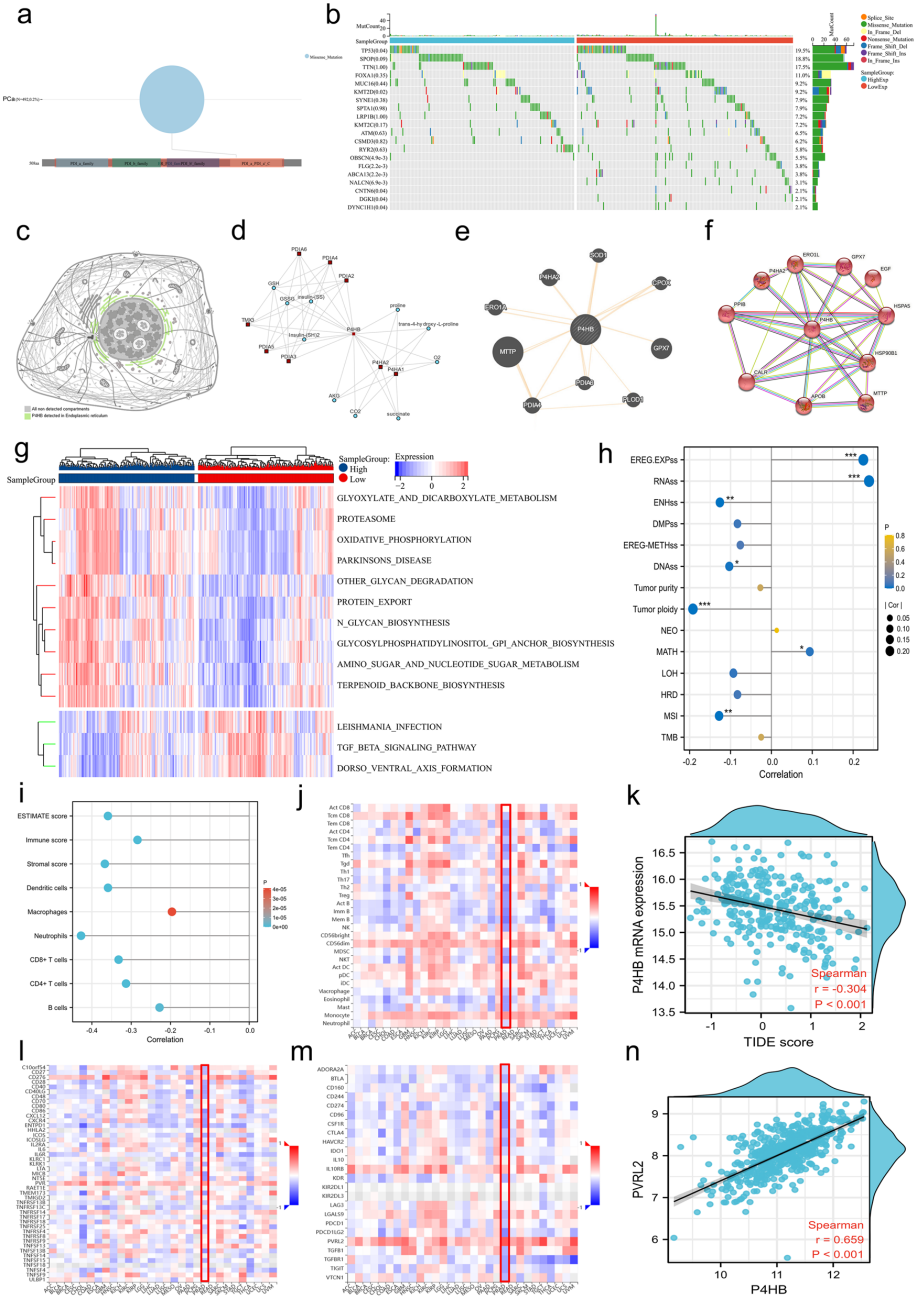
Mutation genes, location, predicted partners, functional pathway, tumor immune microenvironment, tumor heterogeneity and stemness of P4HB. **a** Mutation landscape of PCa patients in the TCGA database; **b** mutation genes between high and low P4HB expression; **c** predicted sublocation of P4HB; **d** reported pathway of P4HB in the HPA database; **e** predicted interaction genes of P4HB using GeneMANIA database; **f** predicted functional partners of P4HB using STRING database; **g** functional pathways between high and low P4HB expression using GSVA methods; **h** bubble diagram showing the correlations of P4HB expression with tumor heterogeneity and stemness; **i** bubble diagram showing the correlations of P4HB expression with tumor microenvironment scores; **j** heatmap showing the relationship between P4HB expression and tumor-infiltrating cells; **k** scatter diagram showing the relationship between P4HB expression and TIDE score; **l** heatmap showing the relationship between P4HB expression and immunostimulators; **m** heatmap showing the relationship between P4HB expression and immunoinhibitors; **n** scatter diagram showing the relationship between P4HB expression and PVRL2. *TIDE* tumor immune dysfunction and exclusion

Missense mutations were found in 0.2% of PCa patients, according to the mutation landscape (Fig. 4a). We divided the PCa patients into two groups according to the median value of P4HB. OBSCN, FLG, AGCA13, NALCN, CNTN6, DGKI, and DYNC1HI were significantly differentially expressed between the two groups among the top 20 altered genes, while TP53 was the most frequently mutant gene in PCa (Fig. 4b). In the HPA database [22, 23], P4HB was found in the endoplasmic reticulum (ER) and was implicated in the metabolism of arginine, proline, insulin, and glutathione (Fig. 4c, d). P4HB was predicted to be potential partner of MTTP, P4HA2 and GPX7 using the GeneMANIA [24] (Fig. 4e) and STRING databases [25] (Fig. 4f). Glyoxylate and dicarboxylate metabolism, proteasome, oxidative phosphorylation, protein export, N glycan biosynthesis, glycosylphosphatidylinositol anchor biosynthesis, amino sugar and nucleotide sugar metabolism and terpenoid backbone biosynthesis were upregulated in the group of high-expression P4HB, while TGF beta signaling pathway and dorso ventral axis formation were upregulated in the group of low-expression P4HB (Fig. 4g).

P4HB expression had statistically significant positive relationships with EREG.EXPss and RNAss, but negative relationships with ENHss and DNAss (Fig. 4h). For tumor heterogeneity, P4HB expression was positively related to MATH, but was negatively associated with tumor ploidy and microsatellite instability. However, the correlation values of P4HB with the above indicators were smaller than 0.3, which indicated that the correlation strength was low. For the overall assessment of TME, we observed that P4HB expression was negatively associated with all parameters, including macrophages, dendritic cells, B cells, CD8+ T cells, CD4+ T cells, neutrophils, stromal score, immunological score and ESTIMATE score (Fig. 4i), which was confirmed by the TISIDB database [31] (Fig. 4j). Spearman analysis showed that P4HB expression was negatively related to TIDE score with statistical significance (Fig. 4k). Similar results were observed in terms of immunostimulators (Fig. 4l) and immunoinhibitors (Fig. 4m) in the TISIDB database [31]. Among these indicators, it was striking that P4HB expression showed significant relationship with PVRL2 expression with good correlation (*r* = 0.659, *p* < 0.001; Fig. 4n).

**Fig. 5:**
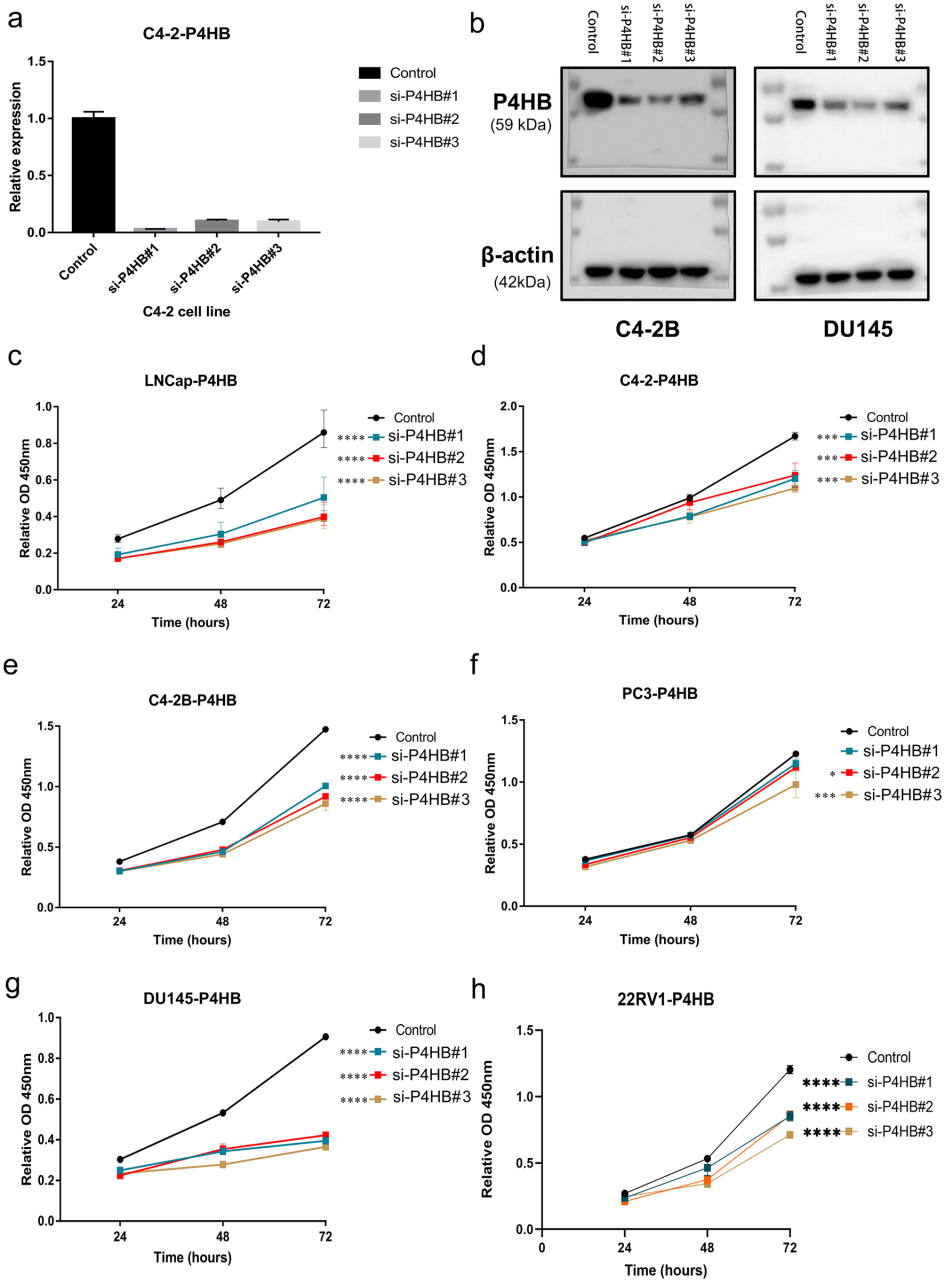
Effect of P4HB on PCa cell proliferation. **a** RT-qPCR results of P4HB siRNAs; **b** western blot results of P4HB siRNAs using C4-2B and DU145 cells; **c** effect of P4HB siRNAs on LNCap using CCK8 assay; **d** effect of P4HB siRNAs on C4-2 using CCK8 assay; **e** effect of P4HB siRNAs on C4-2B using CCK8 assay; **f** effect of P4HB siRNAs on PC3 using CCK8 assay; **g** effect of P4HB siRNAs on DU145 using CCK8 assay; **h** effect of P4HB siRNAs on 22RV1 using CCK8 assay. *RT-qPCR* real-time quantitative polymerase chain reaction

Owing to the opposite results of between prognostic analysis and differential expression and clinical correlations, we detected the impact of P4HB on the cell proliferation of PCa cell lines. RT-qPCR and western blot showed that three siRNAs of P4HB were effective on the knockdown of P4HB expression (Fig. 5a, b). Furthermore, we observed that the downregulation of P4HB had significant influence on the cell proliferation of six PCa cell lines, including LNCap, C4-2, C4-2B, PC3, DU145 and 22RV1 cells (Fig. 5c–h).

## Discussion

Similar to apoptosis, ferroptosis is a form of programmed cell death brought on by the fatal accumulation of iron-dependent lipid peroxides [65–69]. The ferroptosis-related enzyme GSH peroxidase 4 (GPX4) is the only one that can use glutathione (GSH) as an electron donor to remove harmful lipids from biofilms. Glutathione (GSH) can reduce lipid peroxidation to prevent membrane damage [70]. The basic mechanisms of ferroptosis include GSH depletion and decreased GPX4 activity. Another recently found GSH-independent ferroptosis pathway includes Q10 (CoQ10) and CoQ oxidoreductase ferroptosis-suppressing protein 1 (FSP1) [71, 72]. Some ferroptosis inducers, such as erastin and sorafenib, work by inactivating GPX4, while the tiny molecule FIN56 works by depleting GPX4 protein and CoQ10 at the same time [73, 74]. Even tumor cells that have demonstrated resistance to apoptosis are innately susceptible to ferroptosis. For instance, PCa is reliant on mitochondrial metabolism early on and exhibits altered fatty acid production and oxidation pathways, which raises the possibility that ferroptosis may be involved in the carcinogenesis of this disease [27].

The most prevalent cancer in the western world is unquestionably PCa [75]. It is a type of cancer liable to ferroptosis induction, e.g., enzalutamide therapy results in GPX4 inhibition and consequent ferroptosis sensitization [76, 77]. The ferroptosis inducers erastin or RSL3 markedly decreased prostate cancer cell growth and migration in vitro and markedly slowed tumor growth of treatment-resistant prostate cancer in vivo, indicating that this could be used in conjunction with conventional second-generation antiandrogens for PCa treatment [77]. Furthermore, even in PCa that is resistant to castration, PCa cells respond to the ferroptosis inducer erastin [77]. Recent research has revealed that the ferroptosis inducer erastin can both in vitro and in vivo down-regulate the androgen receptor (AR) and its splice variants, which are essential for the development of castration-resistant PCa [78]. Future prospects for PCa therapy may involve combining several ferroptosis inducers with conventional antineoplastic or antiandrogen medications. For instance, the combination of docetaxel and erastin improves the inhibition of castration-resistant PCa by inhibiting the expression of both full-length and splice variants in cancer cells, and the combination of an isothiocyanate-containing hybrid AR antagonist and the GSH synthesis inhibitor buthionine sulfoximine results in ferroptosis and lowers AR activity [78]. Ferroptosis has considerable prospects in PCa tumorigenesis [79] and treatment.

The prolyl 4-hydroxylase beta subunit is encoded by the gene P4HB, which is located at 17q25.3. Preprocollagen's prolyl residues are hydroxylated by P4HB, and this process has the primary effect of preventing the aggregation of improperly folded proteins. For the protein-folding catalyst, bacitracin is regarded as either a selective or nonspecific P4HB inhibitor [80]. P4HB has been reported to be associated with a variety of cancer and oncological outcomes, like bladder cancer from our previous study [81]. Direct deletion of this gene makes cells more susceptible to known ferroptosis inducers, while the P4HB inhibitor PACMA31 directly promotes ferroptosis [82]. The intermediate regulators SLC7A11 and GSH work in concert with a variety of upstream factors, such as many lncRNAs and circRNAs, to control ferroptosis. The expression of circP4HB in lung adenocarcinoma (LUAD) was found to be elevated both in vivo and in vitro, and it was shown to prevent ferroptosis caused by erastin through regulating miR-1184/SLC7A11-mediated GSH production, which promoted tumor growth [83]. In addition, in LUAD cells with significant P4BH expression, the enrichment and positive expression of the GSH metabolic pathway were clearly seen [83]. Our study is the first to describe the predictive role of P4HB in PCa prognosis and speculate its possible mechanism related to ferroptosis. Similar to LUAD, the results of our in vitro experiments showed that P4HB downregulation of multiple PCa cell lines significantly reduced proliferation, and the P4HB high-expression group had significantly higher Gleason score and more advanced T stage.

The majority of protein synthesis takes place in the ER, and the P4HB protein serves as an ER chaperone to ensure that newly generated proteins are folded correctly [84, 85]. The chemicals that influence ferroptosis by altering lipid peroxidation are predominantly located in the ER, which is the most significant organelle for ferroptosis [86]. One of the fundamentals of prostate carcinogenesis, ER stress is a rapidly reproducing cell's adaptive defensive response that frequently manifests in tumor cells [87, 88]. Ferroptosis was discovered to be brought on by the activation of the ER stress signaling system [89, 90]. In these conditions, the requirement for protein synthesis rises, activating the unfolded protein response (UPR) [91, 92]. In PCa, it was discovered that the UPR is androgen-sensitive, and AR signaling controls enhanced protein folding, mRNA degradation, and protein translation, boosting PCa cell survival by blocking the PERK-eIF2a axis [93–95]. During endoplasmic reticulum stress, the chaperones are primarily in charge of facilitating protein folding and removing abnormal proteins [96]. While some chaperone proteins have been discovered to be involved in cancer and drug resistance, ER chaperones are currently not thought to be confined to functions required for protein folding, assembly, and membrane protein transport [97, 98]. Fonseca et al. found that P4HB and other protein disulfide isomerases are immunogenic, and the gene products they produce could be used as therapeutic monoclonal antibody targets [99].

We also discovered that the high-expressing P4HB group had an up-regulated level of energy metabolism, including oxidative phosphorylation, as well as protein production. According to the Warburg effect, it is well known that maintaining tumor metabolism necessitates greater energy supply and metabolic activity and that tumor cells significantly rely on antioxidant systems. For instance, oxidative phosphorylation disruption can raise unstable iron pools and increase the risk of ferroptosis in cells by preventing mitochondrial metabolism [100]. This implies that the main gene of P4HB can be used to particularly increase the ferroptosis sensitivity of malignancies by blocking metabolism.

We discovered through functional analysis that P4HB, which was found in the ER, was involved in GSH metabolism. GSH is a crucial cofactor for the enzyme GPX4 in the conversion of lipid hydroperoxides to lipid alcohols, which reduces lipid peroxidation and prevents ferroptosis [101, 102]. We hypothesized that P4HB might control tumor cell ferroptosis by contributing to GSH depletion. Fibroblasts has been reported to be important to many diseases, including cancers [8, 103–108]. Our previous study also observed that P4HB was related to cancer-related fibroblasts [62]. Apart from this kind of stromal cell, we discovered that P4HB had a negative correlation with a range of immune cells in the tumor microenvironment, such as T cells, B cells, and macrophages. This finding inferred that P4HB functions as a pro-oncogene with immunosuppressive, pro-angiogenic, and anti-inflammatory effects, creating a stromal microenvironment that is favorable for the growth and transformation of prostate epithelial cells, resulting in PCa [109]. However, the causal relationship between immune cells and ferroptosis remains questionable. For instance, interferon gamma (IFN)-mediated ferroptosis of tumor cells is one way that CD8+ T lymphocytes contribute to the suppression of malignancies [110]. T cells have the ability to internalize P4HB, which improves their activation, proliferation, adhesion, and migration [111]. In addition, the novel PDI inhibitor E64FC26 was discovered to alter T cell metabolism and decrease global P4HB expression in healthy T cells, which improves immune responses against tumors [112]. Notably, we found that the P4HB group with low expression triggered the TGF signaling pathway. Inhibiting the TGF signaling pathway enhances the immune response in the TME, which is followed by the polarization of M1-type macrophages, which triggers the Fenton response and the consequent ferroptosis of tumor cells. This provides a possible course of action for the therapy of cancers [113]. PCa patients may benefit from this TGF-β receptor inhibitor and modified nanoparticle breast cancer medication (SB431542) [114]. Thus, there is reason to believe that the immune microenvironment's crosstalk may contribute to the up-regulation of the ferroptosis-related gene P4HB in malignancies.

In addition, even though we were able to demonstrate that si-P4HB has an anti-proliferative effect on six PCa cells, more researches are required to show and understand the role of P4HB in PCa, like overexpressing P4HB in PCa cells as well as in vivo animal studies and so forth. Further research is needed based on our current findings. According to our research, P4HB is a new oncogene connected to the development of prostate tumors. ER stress and modifications to the metabolic route may be related to the process. We hypothesized that one of the anti-cancer targets could be achieved by inhibiting the pro-oncogene P4HB, such as using the P4HB inhibitor bacitracin. This hypothesis has to be verified by subsequent in vitro and in vivo testing. The mechanism of P4HB and ferroptosis in PCa must be identified, as well as if ferroptosis-inducing drugs may be utilized in conjunction with immune checkpoint inhibitors, or whether using ferroptosis to activate immune cells or target metabolic patterns to trigger ferroptosis can help treat PCa.

## Conclusions

In this study, we found that P4HB might serve as a prognostic biomarker and predict radiotherapy resistance for PCa patients. Downregulation of P4HB expression could inhibit the cell proliferation of PCa cells.

## Data Availability

The results showed here are in whole or part based upon data generated by the TCGA Research Network: https://www.cancer.gov/tcga.
